# *Agrobacterium tumefaciens*-Induced Bacteraemia Does Not Lead to Reporter Gene Expression in Mouse Organs

**DOI:** 10.1371/journal.pone.0002352

**Published:** 2008-06-04

**Authors:** Igor V. Petrunia, Olga Y. Frolova, Tatiana V. Komarova, Sergey L. Kiselev, Vitaly Citovsky, Yuri L. Dorokhov

**Affiliations:** 1 A.N. Belozersky Institute of Physico-Chemical Biology, Moscow State University, Moscow, Russia; 2 Department of Biochemistry and Cell Biology, State University of New York, Stony Brook, New York, United States of America; 3 N.I. Vavilov Institute of General Genetics, Russian Academy of Science, Moscow, Russia; University of Melbourne, Australia

## Abstract

*Agrobacterium tumefaciens* is the main plant biotechnology gene transfer tool with host range which can be extended to non-plant eukaryotic organisms under laboratory conditions. Known medical cases of *Agrobacterium* species isolation from bloodstream infections necessitate the assessment of biosafety-related risks of *A. tumefaciens* encounters with mammalian organisms. Here, we studied the survival of *A. tumefaciens* in bloodstream of mice injected with bacterial cultures. Bacterial titers of 10^8^ CFU were detected in the blood of the injected animals up to two weeks after intravenous injection. Agrobacteria carrying *Cauliflower mosaic virus* (CaMV) 35S promoter-based constructs and isolated from the injected mice retained their capacity to promote green fluorescent protein (GFP) synthesis in *Nicotiana benthamiana* leaves. To examine whether or not the injected agrobacteria are able to express in mouse organs, we used an intron-containing GFP (GFPi) reporter driven either by a cytomegalovirus (CMV) promoter or by a CaMV 35S promoter. Western and northern blot analyses as well as RT-PCR analysis of liver, spleen and lung of mice injected with *A. tumefaciens* detected neither GFP protein nor its transcripts. Thus, bacteraemia induced in mice by *A. tumefaciens* does not lead to detectible levels of genetic transformation of mouse organs.

## Introduction

Genetically engineered plants often represent a preferred source of recombinant proteins and biopharmaceuticals for human consumption [1], [2]. In modern plant biotechnology, genetic transformation of plants is usually achieved using *Agrobacterium tumefaciens.* This bacterium is a soil-borne, nonpathogenic for humans microorganism which can transfer its T-DNA into the genomes not only of plants but also of human cultured cells (for review, see [3]–[5]). Wide exploitation of *Agrobacterium* for biotechnological purposes and numerous medical cases of *Agrobacterium* species isolation from bloodstream infections [6]–[17] require the assessment of biosafety-related implications of *Agrobacterium* invasion of mammalian organisms.

We studied whether or not intravenously injected *A .tumefaciens* can survive in mouse bloodstream and direct expression of its T-DNA within mouse organs. Our data indicate that, although *Agrobacterium* persisted in the bloodstream for up to two weeks post injection, it failed to express the reporter GFP gene in such diverse organs as spleen, liver and lung. Thus, *Agrobacterium* induces bacteremia in mice, but does not cause detectible genetic alteration of mouse tissues.

## Results and Discussion

**Figure 1:**
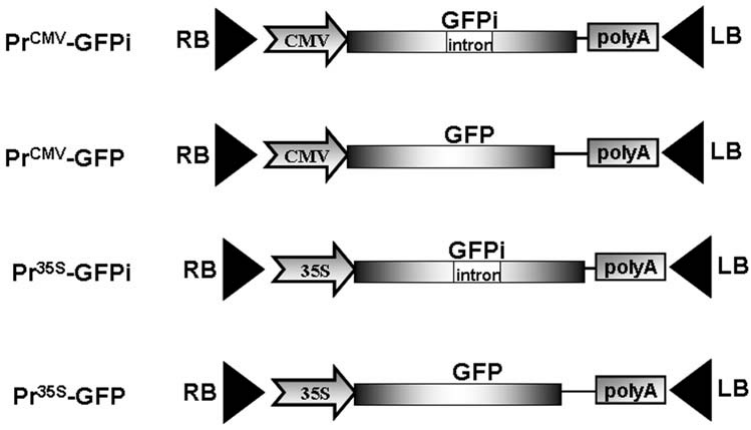
GFP-encoding constructs for intravenous mouse agroinjection. Schematic representation of the cytomegalovirus (CMV) promoter- and CaMV 35S promoter-driven green fluorescent protein cDNA without (GFP) or with an intron sequence (GFPi). All constructs were based on the T-DNA of the pBin19 binary vector. LB and RB indicate the left and right T-DNA borders, respectively. Poly A indicates the CaMV 35S- or CMV-specific transcriptional terminators.

To examine whether agrobacteria can express the reporter gene both in animal and plant cells, we created binary constructs in which GFP expression is driven by either the cytomegalovirus (CMV) or CaMV 35S promoters (Fig. 1). We also inserted a small synthetic intron sequence into the GFP open reading frame (GFPi) to avoid intra-bacterial GFP synthesis due to potentially leaky promoter activity.

**Figure 2:**
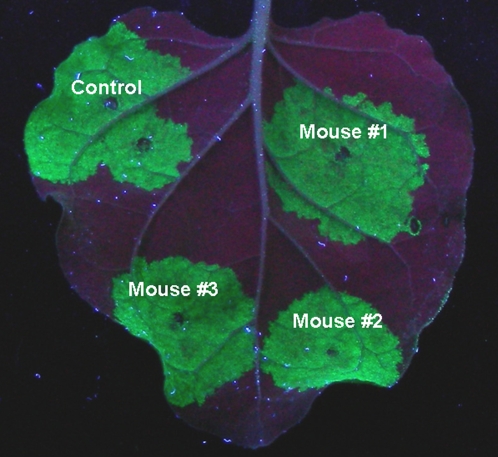
Agrobacteria recovered from the injected mice direct GFP expression in plant cells. GFP accumulation in leaf sectors co-injected with agrobacteria carrying the CaMV 35S promoter-based binary construct and isolated from mouse blood was determined 3 dpi. Control, agrobacteria used for mouse injection.

Agrobacteria carrying these reporter constructs were first characterized for their viability in blood vessel system. To this end, freshly growing *A. tumefaciens* GV3101 (10^8^ CFU) was administered into mouse by tail vein injection, and blood samples were plated on an antibiotic-containing LB agar medium. Table 1 shows that *Agrobacterium* remained viable in the bloodstream during at least 6 days after injection. A few blood samples yielded bacterial colonies even two weeks after injection (data not shown). Agrobacteria contained within the blood samples retained not only the capacity to growth on antibiotic-containing media, but also directed expression of the GFP gene from the CaMV 35S promoter in leaves *Nicotiana benthamiana* (Fig. 2).

**Table 1:** *A. tumefaciens* surviving in mice.

Mouse number	Time after agroinjection	Colony-forming units	
		per plate	average
1	1h	88	89.0
		90	
2		92	131.0
		170	
3		250	158.5
		67	
4		90	82.5
		75	
5		100	67.5
		35	
1	24h	9	8.0
		7	
2		12	8.5
		5	
3		15	10.5
		6	
4		4	3.5
		3	
5		35	23.5
		12	
1	48h	23	17.0
		11	
2		51	44.0
		37	
3		96	59.0
		23	
4		15	50.0
		85	
5		150	99.0
		48	
1	72h	3	8.5
		14	
2		4	4.0
		4	
3		7	5.0
		3	
4		4	8.0
		12	
5		17	18.5
		20	
1	96h	11	47.0
		83	
2		5	16.5
		28	
3		43	43.5
		44	
4		27	22.5
		18	
5		30	33.5
		37	
1	144h	11	32.0
		53	
2		112	74.0
		36	
3		170	148.5
		27	
4		28	23.5
		19	
5		37	26.0
		15	

**Figure 3:**
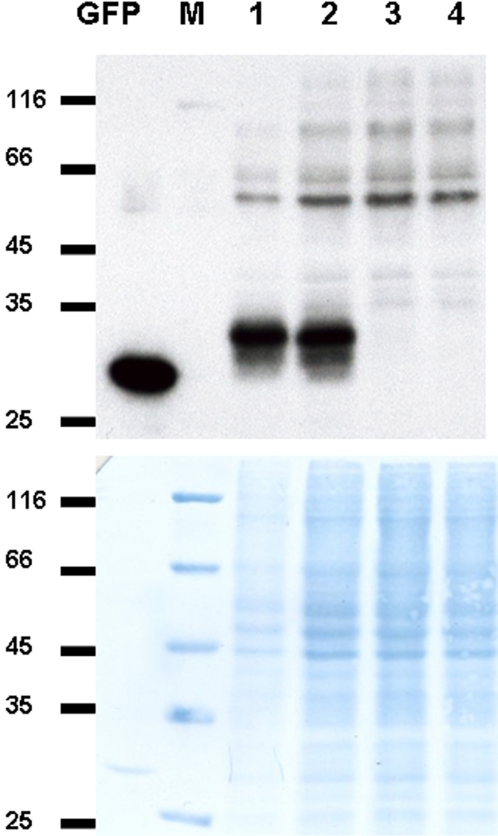
GFP detection in HeLa cells transfected with CMV promoter-based (lanes 1, 2) and CaMV 35S promoter-based (lanes 3,4) constructs encoding GFP (lanes 1 ,3) or GFPi (lanes 2, 4). M, protein molecular weight markers.

Next, we studied the expression of the intron-containing GFPi gene in HeLa cells. On Western blots (Fig. 3), the GFP antibody labeled a 31-kDa band in samples transfected either with CMV-directed GFPi or with CMV promoter-directed GFP (lanes 1, 2). The 27-kDa GFP expressed in mammalian cells is known to assume folding that corresponds to an apparent electrophoretic mobility of a 31 kDa protein [18], [19]. This GFP-specific signal was not observed in samples transfected with CaMV 35S promoter-based constructs (Fig. 3, lanes 3, 4). Thus, mammalian cells promoted the correct splicing of the *Petunia hybrida* intron sequence.

**Figure 4:**
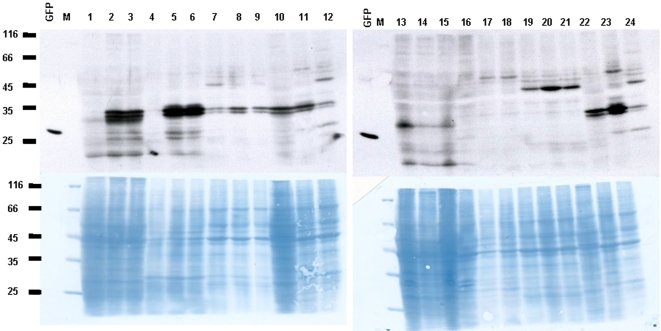
Western blot analysis of protein extracts from different organs of mice injected with agrobacteria. Liver (lanes 1–3, 10, 13–15) after injection with agrobacteria containing the CMV promoter-based vectors encoding GFPi (lanes 1–3, corresponding to mice #1–3) or GFP (lane 10), or an empty vector (lanes 13–15, corresponding to mice #5–7); Spleen (lanes 4–6, 11, 16–18) after injection with agrobacteria containing the CMV promoter-based vectors encoding GFPi (lanes 4–6, corresponding to mice #1–3) or GFP (lane 11), or an empty vector (lanes 16–18, corresponding to mice #5–7); Lung (lanes 7–9,12,19–21) after injection with agrobacteria containing the CMV promoter-based vectors encoding GFPi (lanes 7–9, corresponding to mice #1–3) or GFP (lane 12), or an empty vector (lanes 19–21, corresponding to mice #5–7). Control (lanes 22–24)–liver (lane 22), spleen (lane 23) and lung (lane 24) of an intact mouse. GFP–bacterially expressed 27-kDa GFP (2 ng) as a positive control.

**Figure 5:**
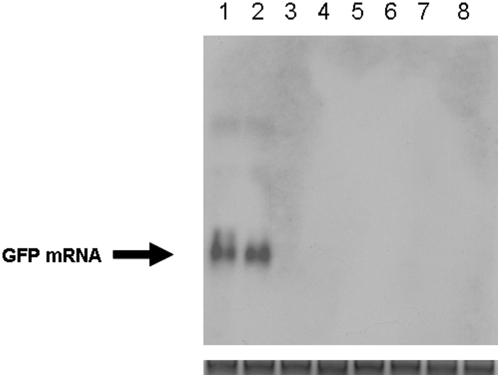
Northern blot analysis of RNA isolated from HeLa cells and mice livers. HeLa cells were transfected with CMV promoter-based (lanes 1, 2) and CaMV 35S promoter-based constructs (lanes 3, 4) encoding GFPi. Mice #1–3 (lanes 6–8) were injected with agrobacteia harboring the CMV promoter-based constructs encoding GFPi. Mouse injected with agrobacteria harboring an empty binary vector (lane 5). Top panel, GFP-specific transcripts; bottom panel, 28S rRNA used as a loading control.

**Figure 6:**
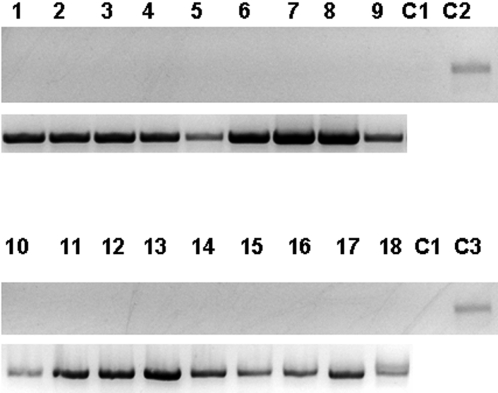
RT-PCR analysis of RNA isolated from liver (lanes 1–3, 10–12), spleen (lanes 4–6, 13–15) and pulmonary (lanes 7–9, 16–18) tissues of mouse injected with agrobacteria harboring CMV promoter-based binary constructs with the GFP (lanes 1–9) or GFPi gene (lanes 10–18). C1, control 1–RNA isolated from a mouse injected with agrobacteria carrying an empty vector; C2, C3 controls 2 and 3–RNA isolated from HeLa cells transfected with the CMV-based constructs containing the GFP or GFPi gene, respectively. Top panel, GFP-specific RT-PCR products; bottom panel, actin-specific RT-PCR products used as a loading control.

Interestingly, our western blot analyses of proteins from different organs of mice injected with bacteria carrying the CMV promoter-drive reporter construct did not revealed 27–31 kDa GFP-specific products, although some protein samples, including those from control, uninjected mice exhibited non-specific cross-reactivity of anti-GFP antibodies with a 35-kDa double-band (Fig. 4). Consistently, northern blot hybridization (Fig. 5) and RT-PCR analysis (Fig. 6) of RNA isolated from different organs of agroinjected mice did not detect any GFP-specific transcripts. These observations indicate that *A. tumefaciens* can persist in the bloodstream of agroinjected mice for relatively long periods of time, but they are unable to cause detectible levels of genetic transformation of mouse organs.

That *A. tumefaciens* and related species can infect mammals, including humans, is supported by numerous medical literature [6]–[17]. However, it should be noted that virtually all the reported cases of such infection occur in immunologically compromised patients who often also have suffered a loss of body integrity, such as invasive surgery, catheter insertion, or a major wound, which presumably allow bacterial access to the bloodstream or internal tissues. Thus, most likely, in healthy individuals, the immune system successfully copes with *A. tumefaciens* which persists in most of our natural environment, e.g., soil, plant roots, etc. Furthermore, that Agrobacterium strains isolated from infected patients often do not contain the Ti plasmid or its elements required for genetic transformation [6]–[17] suggests that (i) the genetic determinants of this opportunistic infectivity likely reside in the bacterial chromosome, rather than in the Ti plasmid, and (ii) unlike infection of plants, infection of mammals by Agrobacterium does not necessitate genetic alteration of the host. This idea is consistent with our observations that *A. tumefaciens* can persist in the mouse bloodstream without detectible expression of its T-DNA in the host tissues as well as with previous findings that *A. tumefaciens* genetically transforms cultured mammalian cells inefficiently [20], probably at the levels undetectable in whole animal tissues. Interestingly, embryonic tissues may be more susceptible to transformation which has been recently reported for sea urchin embryos [21].

## Materials and Methods

### Plasmids used for agroinjection

The CaMV 35S promoter-based plasmid encoding GFP was described earlier [22]. *A. tumefaciens* containing in its T-DNA the *GFP* gene with *Petunia hybrida* PSK7 gene 74-nt intron-7 sequence (GFPi) was kindly provided by Dr. Y. Gleba. For construction of the CMV promoter-based vectors, GFP or GFPi cDNA was inserted into the pcDNA3.1 plasmid digested with XhoI and HindIII. The resulting plasmids were digested with MfeI and SphI, filled-in with the Klenow enzyme and ligated into the binary vector pCAMBIA1300 after its digestion with XhoI and HindIII and filling-in with Klenow.

### Agroinjection procedure

*A. tumefaciens* strain GV3101 was transformed with individual binary constructs, grown in LB medium supplemented with rifampicin 50 mg/l at 28°C. *Agrobacterium* cells from an overnight culture (2 ml) were collected by centrifugation (10 min, 4,500×g), resuspended in saline and adjusted to a final OD_600_ of 0.2. The resulting bacterial suspensions were administered into each recipient naive BALB/c mouse intravenously through the tail vein. For determination of bacterial titers, blood samples (50 µl) from tail vein of mice were added into a tube (950 µl) with heparin (6 units/ml) containing LB growth medium and RGK antibiotic mixture [rifampicin (50 µg/ml), gentamicin (25 µg/ml), kanamycin (100 µg/ml)]. A sample (250 µl) of this mixture was plated on an LB-RGK agar medium, and the bacterial titer was calculated as number of colony forming units (CFU).

### Human cell transfection

HEK293 (human embryonic kidney) cells were plated on 35-mm Petri dishes. For transfection, 2 µg of plasmid DNA and 3 µl of Unifectin56™ (Unifect, Russia) were added to each dish in according to the manufacturer's protocol. GFP fluorescence was detected 24 hours after transfection. Forty eight hours post transfection, cells were trypsinized (HyClone Trypsin), washed with 1× phosphate-buffered saline (PBS) and resuspended in PBS. Then, equal volume of 2× loading buffer (60% glycerol, 5 mM β-mercaptoethanol, 10% SDS, 250 mM Tris-HCl pH 6.8) was added to the cell suspension, and extracts were boiled for 5 min and subjected to electrophoresis on 12% SDS/polyacrylamide gels.

### RNA isolation and Northern blot analysis

Isolation of total RNA was performed using TRI-REAGENT (Molecular Research Center, Inc) using the manufacturer's protocol. Northern blot analysis was performed as described earlier [23].

### Reverse Transcription-Polymerase Chain Reaction (RT-PCR) analysis

RT of extracted RNA was performed using the first-strand cDNA synthesis kit (Promega) according to the manufacturer's instructions. First-strand GFP and actin cDNAs were synthesized using 0.5 µg of RNA and primers GFP-M (5′TTACTTGTACAGCTCGTCCATGCCGAGA3′) and Act-M (5′AGGGTACATGGTGGTGCCGCCAGAC3′), respectively. The cDNAs were then amplified by PCR using primers GFP-P (5′ATGGTGAGCAAGGGCGAGGAGCTGTTC3′) and Act-P (5′CCAAGGCCAACCGCGAGAAGATGAC3′), respectively. PCR was carried out in a programmable Tercik thermocycler (DNA technology, Russia) with the following conditions: 94°C for 10 minutes followed by 30 cycles, each comprising denaturation for 1 minute at 94°C; annealing for 1 minute at 58°C for GFP or 60°C for actin; and then extension for 1 minute at 72°C. After completion of PCR, reaction tubes were kept for 5 minutes at 72°C and then stored at 4°C until use. Negative controls routinely used for each set of primers included reactions without template. Samples were analyzed on 1.5% agarose gels.

### Western blot analysis

The proteins from mouse organs (spleen, liver, lung) were isolated using TRI-REAGENT (Molecular Research Center, Inc) according to the manufacturer's protocol and analyzed by western blotting with GFP-specific antibodies as described earlier [23].
